# The effect of duration and time preference on the gap between adult and child health state valuations in time trade-off

**DOI:** 10.1007/s10198-023-01612-8

**Published:** 2023-07-08

**Authors:** Zhongyu Lang, Arthur E. Attema, Stefan A. Lipman

**Affiliations:** 1 Erasmus Centre for Health Economics Rotterdam (EsCHER), Erasmus School of Health Policy and Management (ESHPM), Erasmus University, P.O. Box 1738, 3000 DR Rotterdam, The Netherlands; 2 Erasmus School of Health Policy and Management, Erasmus University, Rotterdam, The Netherlands; 3 Erasmus Centre for Health Economics Rotterdam, Rotterdam, The Netherlands

**Keywords:** EQ-5D-Y, Time preference, QALY model, Time trade-off, I10

## Abstract

Composite time trade-off (cTTO) utilities have been found to be higher when adults value health states for children than for themselves. It is not clear if these differences reflect adults assigning truly higher utilities to the same health state in different perspectives, or if they are caused by other factors, which are not accounted for in the valuation procedure. We test if the difference between children’s and adults’ cTTO valuations changes if a longer duration than the standard 10 years is used. Personal interviews with a representative sample of 151 adults in the UK were conducted. We employed the cTTO method to estimate utilities of four different health states, where adults considered states both from their own and a 10-year-old child’s perspective, for durations of 10 and 20 years. We corrected the cTTO valuations for perspective-specific time preferences in a separate task, again for both perspectives. We replicate the finding that cTTO utilities are higher for the child perspective than for the adult perspective, although the difference is only significant when controlling for other variables in a mixed effects regression. Time preferences are close to 0 on average, and smaller for children than adults. After correcting TTO utilities for time preferences, the effect of perspective is no longer significant. No differences were found for cTTO tasks completed with a 10- or 20-year duration. Our results suggest that the child–adult gap is partially related to differences in time preferences and, hence, that correcting cTTO utilities for these preferences could be useful.

## Introduction

The valuation of health states is an important prerequisite for the implementation of health economic evaluations of new drugs and medical treatments. Researchers are showing increasing interest in extending this methodology to valuing children’s health states [1, 2]. A separate instrument has been developed for this purpose by the EuroQol Group, known as EQ-5D-Y-3L [3, 4], for which a valuation protocol has been published recently [5]. The EQ-5D-Y-3L instrument describes health according to 5 dimensions: mobility, looking after oneself, doing usual activities, pain, or discomfort, and feeling worried, sad, or unhappy. Each of them includes 3 levels of severity (level 1 indicating no problems, level 2 some problems, and level 3 a lot of problems). For example, someone with some problems walking about, no problems with looking after oneself, some problems with doing their usual activities, a lot of pain or discomfort, and not feeling worried, sad, or unhappy, is classified as being in health state 21231.

The EQ-5D-Y-3L classification system has been widely used in measuring children’s health states [6–8]. Yet, an area of ongoing discussion is the perspective that is used for valuation of EQ-5D-Y-3L health states [9, 10]. Its valuation protocol asks adult respondents to value health states considering the life of a 10-year-old child, rather than adults valuing hypothetical health states for themselves, which has been conventional for other EQ-5D instruments. Note that, henceforth, we will refer to these two perspectives as *child perspective* and *adult perspective*.

The EQ-5D-Y-3L valuation protocol recommends the use of the time trade-off (TTO) method to assess utilities in the EQ-5D-Y-3L instrument (as well as discrete choice experiments). The TTO method elicits utilities for health states by asking respondents how many years in full health is equivalent to 10 years in a specified imperfect health state, according to the EQ-5D-Y-3L. The corresponding utility of this health state is then estimated to be equal to y/10, with y being the number of years in full health making the respondents indifferent.

Recent work has found differences in TTO utilities for the same health states when valued from different perspectives [11, 12]. In particular, some studies found that TTO utilities elicited with adult perspectives are lower than those elicited with child perspectives [11, 13]. However, current evidence is not very robust. Some studies reported no or only a small difference [14–16], while another study found differences in both directions [17]. Although collectively these studies clearly suggest effects of perspectives may occur, it is unclear why. One explanation for a perspective effect may be the unrealistically short life duration of 10 years of the TTO task. That is, the 10 years in imperfect health (followed by death) respondents are asked to consider imply a large reduction in lifespan compared to the actuarial life expectancy of most adult respondents and the more so for children. Earlier work for adults has shown that beliefs about life expectancy [18–20] and the importance assigned to longevity may explain the reluctance to trade life duration, and Lipman [21] explored if such beliefs also affect TTO utilities elicited with child perspectives but found little to no evidence. The motivation of the present study was to explore the effect of TTO durations more directly, by extending the life duration considered in both perspectives by 10 years. In absolute terms, such an extension in life duration is equal in both perspectives. Yet, the extension in life duration is (proportionally) much larger in a child perspective than when adults value their own health. For example, for a 40-year-old adult, 10 extra years in a TTO task are an increase equal to 25% of their current age, whereas for a 10-year-old child, the extension equals 100%. In this study, we explore if these differences in relative life extensions yield differential effects in adult and child perspectives.

There is substantial evidence that utilities obtained with a TTO task depend on the gauge duration used, implying a violation of the constant proportional trade-off (CPTO) property [22], albeit there is mixed evidence on the direction of this relation. Some studies found utilities to be increasing with a longer duration [23, 24], others found a decreasing [25–28], or mixed pattern [29], while still others did not find a violation of CPTO [30, 31]. These studies, however, have all been performed from the adult perspective. Predicting the exact direction on TTO utilities elicited with a child perspective is therefore not straightforward. For example, extending the duration of TTO tasks by 10 years allows respondents to trade-off more years whilst still making sure children reach adulthood and, hence, might make this perspective more comparable to the adult perspective for longer durations. However, when extending durations in TTO, it is important to consider disadvantages of longer durations. One important disadvantage is that longer durations introduce more potential for distortion by time preferences [31–34]. This distortion need not be equal between adult and child perspectives: some studies found time preferences for someone else’s health or money to differ from time preferences for our own health or money [16, 35, 36].

The aims of our study are therefore to investigate how duration and time preferences affect (the difference between) TTO valuations with child and adult perspectives. To this end, we elicit health state utility by means of a TTO task using two durations, i.e., the standard 10-year timeframe, and a longer timeframe of 20 years for both perspectives. In addition, we estimate time preferences for both these perspectives, and we use these estimates to investigate the effect of perspective-specific time preferences on TTO utilities.

## Method

### Time tradeoff method

We denote a chronic health state q that lasts for t years by (t,q). The TTO method assigns a utility u(q) to q by asking a respondent to compare x years in q to y years in full health (FH), where x is usually set equal to 10. TTO involves a series of choices through which we search the value for y such that (q,x) ~ (FH,y), where ~ denotes indifference. According to the general QALY model [37], this indifference is represented as follows:

1 $${\text{H}}\left( {\text{q}} \right)*{\text{L}}\left( {\text{x}} \right) = {\text{ H}}\left( {{\text{FH}}} \right)*{\text{L}}\left( {\text{y}} \right).$$

Here, L(t) is the utility of life duration, and H(q) is the utility of health state q. The common scaling for H(q) is to set H(FH) = 1, and for L(t) to set L(0) = 0 and L(T) = 1, with T the final period under consideration. Solving for H(q) yields:

2 $${\text{H}}\left( {\text{q}} \right) = {\text{L}}\left( {\text{y}} \right)/{\text{L}}\left( {\text{x}} \right).$$

If someone prefers immediate death over (q,x), then the health state is classified as worse than dead (WTD). This requires a modified TTO approach, and in valuation of EQ-5D instruments typically the composite TTO (cTTO) is used for this purpose [34]. In this procedure, WTD health states are valued by adding 10 years in full health to the 10 years in state considered WTD (i.e., 10 years lead-time). More generally, this entails that the x years in q are preceded by a lead time of z years in FH [38]). The indifference (FH,z;q,x) ~ (FH,y) obtained by this procedure is evaluated by:

3 $${\text{H}}\left( {\text{q}} \right) = \left[ {{\text{L}}\left( {\text{y}} \right) - {\text{L}}\left( {\text{z}} \right)} \right]/\left[ {{\text{L}}\left( {{\text{x}} + {\text{z}}} \right) - {\text{L}}\left( {\text{z}} \right)} \right].$$

In case of the linear QALY model, L(t) = t, and Eq. (2) reduces to H(q) = y/x while Eq. (3) becomes H(q) = (y–z)/x. In the typical cTTO task with a 10-year duration and a 10-year lead time for WTD states, the linear QALY model implies y/10, and (y-10)/10 for better than dead and WTD states, respectively. In this study we consider an extension of cTTO by 10 years, whilst maintaining the 10-year lead-time, which gives: y/20 and (y-10)/20, respectively. The duration of 20 years was chosen to be a substantial increase compared to 10 years, while still being a realistic life expectancy for most respondents in a general public sample. Moreover, we opted for a fixed duration within the entire sample instead of an individual-specific gauge duration, such as the respondent’s subjective or actuarial life expectancy, because the latter would create a lot of heterogeneity, making the results harder to compare.

In order to have a fair comparison between the durations, the 10- and 20-year TTOs would need to have the same utility range of −1 to + 1; therefore, a lead time of 20 years would have to be used in the 20-year TTO, which would result in a total horizon of 40 years. Because this is unrealistic for part of the general public, we instead use a 10-year lead time for the 20-year TTO as well. This means the lowest attainable (uncorrected) utility for this task is − 0.5, vs. − 1.0 for the 10-year TTO (i.e., if one would still prefer immediate death to living 10 years in full health followed by 20 years in health state X, the cutoff value for the uncorrected TTO weight would result from: 10*1 + 20*X < 0, so X would be set to X = −10/20 = −0.5). Still, we think that the benefits of more realism outweigh the costs in terms of decreased comparability, since the use of a 10-year lead time in both tasks increases similarity in the WTD task. To test the effect of these different ranges, we perform a robustness analysis where all utilities of the 10-year condition are censored at −0.5.

### Time preference

**Fig. 1:**
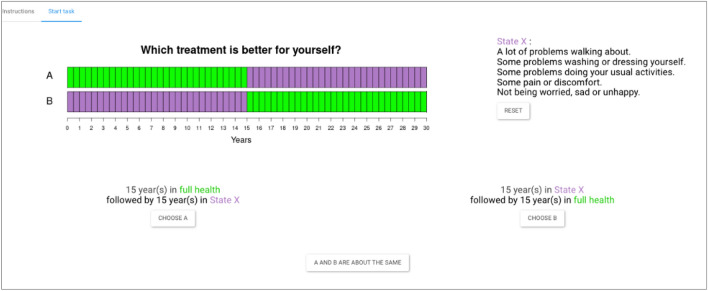
Screenshot of a time preference task

In order to estimate H(q) from Eq. (2), we first need a measure of L(t) or make assumptions about its shape. We use the direct method [39] for this purpose, which has been used to measure time preferences in the context of TTO in several previous studies [40–44]. The advantages of this method are that it is not distorted by risk, does not need to make parametric assumptions about the shape of the discount function, and that it uses a similar context as a TTO task (i.e., quality-of-life improvements, for which we can use the same health states as in the TTO task) [39]. In this method, respondents are asked to compare two health profiles, each consisting of the same two health states, but experienced in a different order. One profile (A) starts with a good health state (γ) and ends with a poorer health state (β), whilst the other profile (B) starts with the poorer health state and ends with good health. The starting and ending periods of the health profiles are identical (T = 30 years), as is the period in which the health state changes. We used a total timeframe of 30 years because it was the maximum duration in the 20-year TTO task (i.e., in the case the WTD procedure with a 10-year-lead time was started). Figure 1 illustrates the task by means of a screenshot of one of the questions in this task. In Profile A, the respondent first lives in full health for 15 years, followed by 15 years in State X. In Profile B, the order of these states is reversed and the respondent first lives in State X for 15 years, followed by 15 years in full health. The respondents were instructed that after this total of 30 years, there was no difference between the two profiles anymore, but the state itself was not specified.

Intuitively, respondents must trade off the onset of the poor health state with its duration. In case of positive discounting, an individual will prefer to start with the good health state and postpone the poor health state. Hence, such an individual will choose Profile A in the first question. In the next question, the amount of time spent in full health is then lowered in Profile A, say to 8 years, whilst the amount of time spent in poor health that follows afterward increases automatically (to 30 − 8 = 22 years). The reverse happens for Profile B, where the amount of time spent in poor health decreases to 8 years and the amount of time spent in full health increases toward 22 years. As such, Profile B has become more attractive, and even respondents with a positive discount rate may prefer it now. Only those respondents with a sufficiently high discount rate keep on preferring Profile A because of its earlier onset of the episode in full health (and, equivalently, the later onset of the episode in poor health). As elaborated further in the discussion section, drawbacks of this method are, inter alia, that it may be distorted by a sequence effect, which holds that respondents could be inclined to choose Profile B because they do not like the anticipation of a decline in their health in the future. Additionally, as in most other methods, respondents could use some heuristics, such as maximizing the time spent in full health.

In formal terms, Profile A is denoted by ([t_0_,t_0.5_], γ; [t_0.5_ + 1,T], β) and Profile B is denoted by ([t_0_,t_0.5_], β; [t_0.5_ + 1,T], γ), where t_0_ is the starting point of the considered episode (year 0 in Fig. 1) and T is the end point (year 30). The time point t_0.5_ is looked for, such that the respondent is indifferent between the two profiles: ([t_0_,t_0.5_], γ; [t_0.5_ + 1,T], β) ~ ([t_0_,t_0.5_], β; [t_0.5_ + 1,T], γ). In the general QALY model, this indifference is represented as follows:

4 $$\left[ {{\text{L}}\left( {{\text{t}}_{0.{5}} } \right) - {\text{L}}\left( {{\text{t}}_0 } \right)} \right]*{\text{H}}(\gamma ) \, + \, \left[ {{\text{L}}\left( {\text{T}} \right) - {\text{ L}}\left( {{\text{t}}_{0.{5}} } \right)} \right]*{\text{H}}(\beta ) \, = \, \left[ {{\text{L}}\left( {{\text{t}}_{0.{5}} } \right) - {\text{L}}\left( {{\text{t}}_0 } \right)} \right]*{\text{H}}(\beta ) \, + \, \left[ {{\text{L}}\left( {\text{T}} \right) - {\text{L}}\left( {{\text{t}}_{0.{5}} } \right)} \right]*{\text{H}}(\gamma ).$$

This equation can be rearranged into:

5 $$\left[ {{\text{L}}\left( {{\text{t}}_{0.{5}} } \right) - {\text{L}}\left( {{\text{t}}_0 } \right)} \right]*\left[ {{\text{H}}\left( \gamma \right) - {\text{H}}\left( \beta \right)} \right] \, + {\text{ L}}\left( {\text{T}} \right)*{\text{H}}\left( \beta \right) \, = \, \left[ {{\text{L}}\left( {\text{T}} \right) - {\text{L}}\left( {{\text{t}}_{0.{5}} } \right)} \right]*\left[ {{\text{H}}\left( \gamma \right) - {\text{H}}\left( \beta \right)} \right] \, + {\text{ L}}\left( {\text{T}} \right)*{\text{H}}\left( \beta \right).$$

Given our scaling of L(t_0_) = 0 and L(T) = 1, this can be simplified into:

6 $${\text{L}}\left( {{\text{t}}_{0.{5}} } \right) = { 1} - {\text{L}}\left( {{\text{t}}_{0.{5}} } \right).$$

Hence, H(γ) and H(β) drop from the equation, and we can estimate the value of t_0.5_ for which L(t) = 0.5, without needing to know H(q).

We can proceed with this elicitation by using the estimate of t_0.5_ in a follow-up question. Specifically, we can elicit t_0.25_ for which L(t_0.25_) = 0.25, such that the respondent is indifferent between the profiles ([t_0_,t_0.25_], γ; [t_0.25_ + 1,t_x_], β) and ([t_0_,t_0.25_], β; [t_0.25_ + 1,t_0.5_], γ), or we can elicit t_0.75_ for which L(t_0.75_) = 0.75, such that the respondent is indifferent between the profiles ([t_0.5_,t_0.75_], γ; [t_0.75_ + 1,T], β) and ([t_0.5_,t_0.75_], β; [t_0.75_ + 1,T], γ), or both. In the first case, we obtain the equation L(t_0.25_) = 0.5 − L(t_0.25_) = 0.25, and in the second case we obtain L(t_0.75_) − 0.5 = 1 − L(t_0.75_), so L(t_0.75_) = 0.75. One can continue this way to get a measurement of L(t) up to any desired degree of precision. In our study, as described below, we elicited the following five points of the discount function: L(t_0.125_) = 0.125, L(t_0.25_) = 0.25, L(t_0.5_) = 0.5, L(t_0.75_) = 0.75, and L(t_0.875_) = 0.875.

## Experiment

### Design and participants

After elaborate pilot testing with students and university staff, who were not part of the formal study, personal interviews were conducted with 151 respondents. We aimed to recruit a sample representative of the English adult population in terms of age, gender, and education. Respondents were recruited by a survey company (Dynata) and received a reward in terms of an addition to their panel points, equivalent to about £30, which could for instance be exchanged into a gift voucher. One of the co-authors (ZL) administrated interviews by using videocalls on Zoom or Google Meet. Only the language of English was used during the whole interview. Participants could complete the designed tasks by following the written steps with the interviewer on the shared screen that was controlled by the interviewer. Any questions could be asked during the interview, which lasted for a maximum of 1 h. The video calls were not recorded for privacy reasons. Ethical approval for this study was provided by the Research Ethics Review Committee of Erasmus School of Health Policy & Management.

#### Interview procedure

The experiment started by participants completing the EQ-5D-Y-3L instrument to allow them to familiarize themselves with its descriptive system. Before the cTTO task, respondents received a cTTO warm-up task featuring the health state “being in a wheelchair”. The interviewer used this example to explain the cTTO task and how their choices would invoke two scenarios: better than dead and worse than dead. After this, two more practice tasks were presented. One of them involved a severe health state that was included expecting it could be considered WTD by many respondents, providing more practice with the WTD procedure included in cTTO.

#### TTO operationalization

TTO was operationalized in 2 blocks, one with a 10-year duration and one with a 20-year duration, which were presented in random order. We completed 4 blocks of TTO tasks (2 perspectives, 2 durations) for 4 health states in a computer-instructed setting. We selected the following health states: 22222, 32211, 32223 and 23232, where the first health state means moderate problems in all 5 dimensions, etc. These health states were also incorporated in Kreimeier et al. [11] and cover a wide spectrum of severity.

We implemented the EQ-VT protocol [45, 46], with the standard time horizon logically changed from 10 to 20 years for the 20-year task. The EQ-VT protocol involved a bisection procedure for the first three steps followed by upward/downward titration with 1-year or 6-month increments. For the 20-year task, an extension of the standard cTTO task in the EQ-VT protocol was developed by MathsinHealth (a consulting firm which is an expert in health economics research).

#### Time preference measurement

Health states β = 32211 and γ = 11111 were used to serve as the respective bad and good health states in the time preference task, from both the adult and child perspective. The corrected TTO utilities were computed by applying this discounting information to the TTO answers, using linear interpolation if a TTO answer was between two points on the discount function. For example, suppose someone values 7 years in full health the same as 20 years in health state 32211. From the discounting task, we have elicited t_0.125_ = 3, t_0.25_ = 6, t_0.5_ = 14, t_0.75_ = 21 and t_0.875_ = 24 for this respondent. Then we estimate L(7) to be 0.25 + (7 − 6)/(14 − 6)*(0.5 − 0.25) = 0.281 and L(20) to be 0.5 + (20 − 14)/(21 − 14)*0.25 = 0.714. Applying Eq. (2) then gives h(32211) = 0.281/0.714 = 0.394. Note that without correcting for discounting we would obtain h(32211) = 7/20 = 0.35. Details about correcting TTO utilities for discounting with the Direct Method can be found in Attema et al. [41]. The task was programmed using software in Shiny.^1^

The framing in the child perspective part of the Direct Method was similar as in the TTO task. That is, the respondents had to consider health improvements for a 10-year-old child. In the first question, they would for example choose between a direct health improvement to full health for the child for the next 15 years, followed by state 32211 in the subsequent 15 years, and a postponed health improvement. The latter would entail the child first living in state 32211 for 15 years, followed by 15 years in full health.

The order of the blocks was randomized, as well as the order of the tasks within the TTO and time preference blocks and the order of the health states within the TTO tasks.

### Analysis

#### Data quality

A data quality check was performed for all 4 TTO tasks. This included the number of non-trading responses (i.e. h(q) = 1), the number of all-in-trading responses (i.e. h(q) = -1 for the 10-year-task and h(q) = −0.5 for the 20-year-task), the number of responses implying a state was valued the same as death (i.e. h(q) = 0), and the number of respondents per task who valued all health states the same [47]. Furthermore, we could perform some dominance tests, because state 22222 is strictly better than states 32223 and 23232, and state 32211 is strictly better than 32223. For example, if weak dominance holds, we should have h(22222) ≥ h(32223) and strict dominance would imply h(22222) > h(32223). We counted the number of weak and strict dominance violations for all these 3 health state pairs.

#### Utilities

We compare the TTO utilities between the perspectives for all 4 health states and 2 durations, using paired t-tests. Second, we compute the differences between the utilities obtained from the adult and child perspective for all health states and perform paired *t*-tests that compare these gaps for the 10- and 20-year durations. Finally, we compare these gaps for the uncorrected and the corrected TTO utilities, again performing paired t-tests.

#### Time preferences

We also compare the discount functions obtained from the two perspectives. This is captured by the ‘area-under-the-curve’ (AUC) approach [48–52]. Because of our normalization, this area is bounded between 0 and 1, and a value of AUC of 0.5 equals zero discounting, i.e., no time preferences. AUC > ( <) 0.5 indicate positive (negative) discounting. As such, someone who has AUC > 0.5 considers years in the future to have less value than years today, whereas the opposite holds for AUC < 0.5.

#### Mixed effects regressions

Finally, we perform mixed-effects regressions of both the uncorrected and the corrected TTO utilities, with subject random effects and dummies for perspective, duration, and health states, as well as several socio-demographic variables:

7 $${h}_{i,q}=\alpha +{HS}_{q}{\beta }^{^{\prime}}+\gamma {D}_{20}+\delta {P}_{C}+{{x}_{i}\gamma }^{^{\prime}}+\varepsilon .$$

In this model, ${h}_{i,q}$ are the utilities, ${HS}_{q}$ is a matrix containing the health state dummies, D_20_ is a duration dummy taking value 1 for the 20-year task, and P_C_ denotes a perspective dummy taking value 1 for the child perspective. Furthermore, ${x}_{i}$ is a matrix containing the other variables (gender, age, own health rating, education, children, religion, subjective life expectancy of children and adults), $\alpha$ is a constant reflecting the adult perspective of the 10-year task valuing health state 22222, and $\varepsilon$ is an error term.

## Results

### Sample description

The sample is summarized in Table 1 below and is reasonably representative of the UK adult public in terms of age, gender, and education, with a slight overrepresentation of highly educated respondents. According to the summary of the UK census in 2020, 23.34% fall under the age of 19, 26.14% are aged between 19 and 39, 31.87% belong to the 40 to 65 age group, and 18.65% are over 65 years; females and males account for 51% and 49% of the whole population, respectively. By the year 2020, among individuals aged from 25 to 64 years, 18.3% had education level below the upper secondary, 32.3% had finished upper secondary or post-secondary non-tertiary education, while 49.4% had completed tertiary education, which includes short-cycle tertiary education, bachelor’s or equivalent, master’s or equivalent and doctoral or equivalent [53].

**Table 1:** Summary statistics

Variables	Percentage	Mean	SD
Age		51.6	15.7
19–39	29.1%		
40–65	45.7%		
65+	25.2%		
Gender			
% Male	48.3		
% Female	51.7		
% Other	0		
Education			
Lower	20.5		
Middle	21.9		
Higher	57.6		
Health status: VAS		79.4	14.3
Expected age of own death		83.7	8.4
Expected age of death of child of 10 years		87.5	9.3
Having children	61.6		
Being religious	27.8		

Low education: elementary school or pre-vocational secondary education; middle education: secondary vocational education or upper-level secondary school); high education: higher professional education or university

### Data quality

Table 2 gives some statistics related to data quality. The results indicate that respondents give more non-trading responses (h(q) = 1) for children (10y: 18.9%; 20y: 15.1%) than for adults (10y: 13.8%; 20y: 14.1%) under both conditions, which is statistically significant for the 10-year condition (binomial proportion test: *p* < 0.02), but not for the 20-year condition (*p* = 0.62). The other comparisons between adult and child tasks were not significant at the 5% level. Comparing the 2 durations, we find some evidence for more non-trading for the 10-year variant than for the 20-year variant. A binomial test for proportions shows significance at the 10% level for children (*p* = 0.079), but not for adults (*p* = 0.87). There is no evidence suggesting that the increased duration affects all-in-trading responses (sacrificing all 10 years of lead-time, *p* > 0.33). There were also no significant differences for the number of h(q) = 0 values (*p*'s > 0.30), the number of respondents that value all states the same (*p*'s > 0.11), and the proportion of dominated responses (*p*'s > 0.17).

**Table 2:** Data quality for both durations of adult and child perspectives

Categories	TTO (10y)-Adult	TTO (10y)-Child	TTO (20y)-Adult	TTO (20y)-Child
Responses without trading (h(q) = 1) (out of 604 observations)*	83 (13.7%)	114 (18.8%)	85 (14.1%)	91 (15.1%)
All-in trading responses (h(q) = −1/−0.5) (out of 604 observations)*	12 (2.0%)	11 (1.8%)	8 (1.3%)	16 (2.6%)
Responses implying zero trading h(q) = 0 (out of 604 observations)*	19 (3.1%)	21 (3.5%)	19 (3.1%)	15 (2.5%)
All states valued the same (out of 151)**	12 (7.9%)	11 (7.3%)	7 (4.6%)	14 (9.3%)
Respondents without 0.5-year increments (out of 151)	78 (51.7%)	82 (54.3%)	100 (66.2%)	96 (63.6%)
Weak dominance violation (e.g., h(q)(22222) < = h(q)(32223), h(q)(22222) < = h(q)(23232)) (out of 453)^†^	139 (30.7%)	140 (30.9%)	114 (25.2%)	132 (29.1%)
Strict dominance violation (e.g., h(q)(22222) < h(q)(23232), h(q)(22222) < h(q)(32223)) (out of 453)^†^	34 (7.5%)	29 (6.4%)	33 (7.3%)	31 (6.8%)

^*^151 respondents × 4 health states

^**^151 respondents

^†^151 respondents × 3 health state pairs

### Time preference

**Fig. 2:**
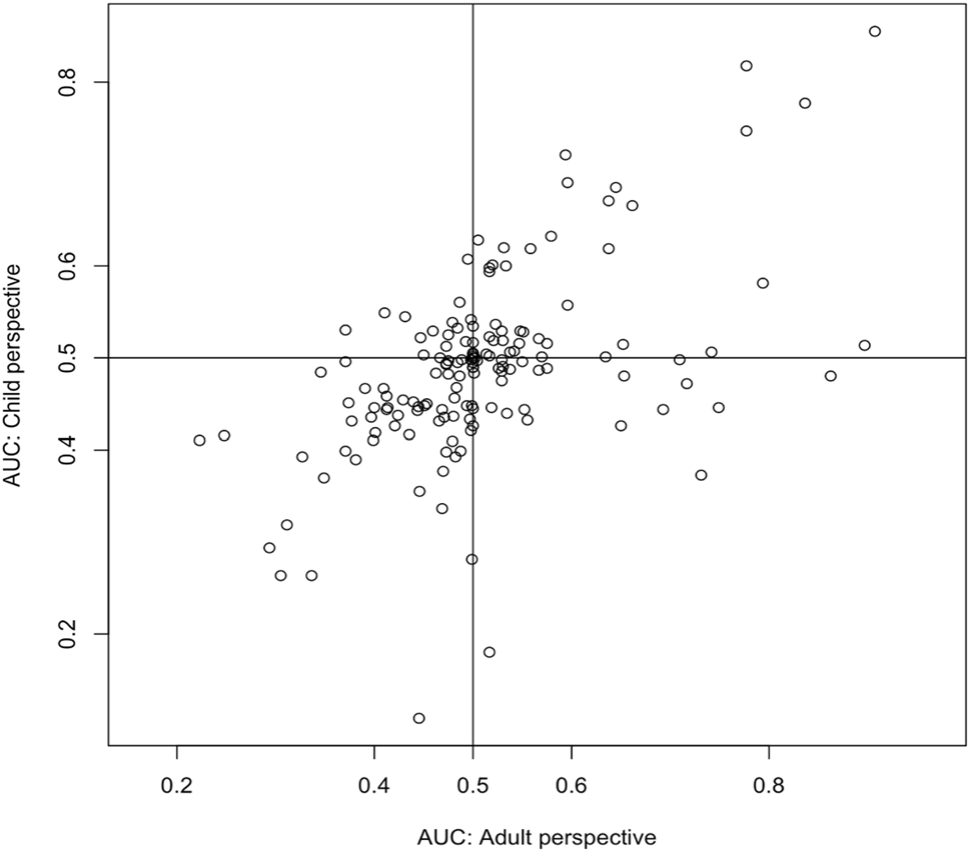
Scatterplot of area-under-the-curve (AUC) for the adult and child perspectives

Figure 2 plots the AUC derived from the direct method completed with a child- or self-perspective, within-subjects. This scatterplot indicates large heterogeneity of time preferences. Furthermore, we find that AUC for children is slightly smaller than for adults, but the difference is not significant (means: 0.502 (Adults), 0.489 (Children); paired *t*-test: *p* = 0.14). Both AUCs are not significantly different from 0.50 (*t* test: *p*’s > 0.11).

We also classified respondents according to their time preferences and determined if their AUC was larger for adult or child perspectives, as shown in Table 3 below. Sixty out of 151 respondents (39.7%) discounted negatively for both children and adults, compared with 39 out of 151 respondents (25.8%) who discounted positively.

**Table 3:** Classification of respondents according to their time preferences

Children	Adults		
	Positive discounting	No discounting	Negative discounting
Positive discounting	39	2	24
No discounting	5	2	5
Negative discounting	14	1	60

### TTO utilities

We investigated the mean uncorrected and corrected utilities for all health states, by perspectives and conditions, as presented in Table 4. It is clear from this table that health states are valued higher from the child perspective than from the adult perspective, with the former perspective yielding higher mean utilities for all 4 health states in both durations. However, this difference is not significant for any of the comparisons (paired *t*-tests, all *p*’s > 0.07 for the uncorrected utilities and all *p*’s > 0.30 for the corrected utilities).

**Table 4:** Mean TTO utilities (standard deviations in parentheses)*

Health state	10-year						20-year					
	Adult		Child		Sig. adult versus child		Adult		Child		Sig. adult versus child	
	U	Cor	U	Cor	U	Cor	U	Cor	U	Cor	U	Cor
22222	0.7 (0.35)	0.71 (0.39)	0.74 (0.32)	0.72 (0.42)	0.08	0.61	0.71 (0.32)	0.72 (0.32)	0.73 (0.3)	0.71 (0.36)	0.21	0.70
32111	0.73 (0.31)	0.74 (0.31)	0.76 (0.31)	0.76 (0.3)	0.19	0.31	0.71 (0.31)	0.72 (0.31)	0.75 (0.31)	0.73 (0.37)	0.08	0.71
32223	0.52 (0.42)	0.54 (0.41)	0.55 (0.45)	0.54 (0.49)	0.29	0.95	0.52 (0.36)	0.54 (0.36)	0.56 (0.38)	0.53 (0.42)	0.13	0.75
23232	0.45 (0.46)	0.47 (0.49)	0.49 (0.47)	0.47 (0.56)	0.20	0.93	0.51 (0.37)	0.52 (0.39)	0.52 (0.38)	0.49 (0.45)	0.69	0.37

^*^U = uncorrected; Cor = corrected

Correction for time preference had little effect on mean utilities. Out of the 16 observations (4 health states, 2 perspectives, 2 conditions), there were only 2 states for which we found evidence that correcting for time preference yielded significant differences in utilities. When using non-parametric tests, slightly more evidence is observed, i.e., paired Wilcoxon tests are significant for 3 states. Still, the between-perspective difference has decreased, and the *p*-values have correspondingly increased. It is also noteworthy that the corrected utilities are lower than the uncorrected utilities for the child perspective, due to negative average time preference, while the opposite holds for the adult perspective. Consequently, the perspective gap decreases after correction for time preference.

There is also no evidence in favor of significant differences between the 10- and 20-year duration, neither for the adult, nor for the child perspective (all *p*’s > 0.17, except for state 23232 with higher utility for 20 years than 10 years under the adult perspective, *p* < 0.01). These results were similar when using the censored 10-year TTO (all *p*’s > 0.50).

Finally, we do find several significant differences when comparing the adult–child gaps for the uncorrected utilities with the adult–child gaps for the corrected utilities. In particular, the gap is lower for the corrected utilities for all 8 comparisons, with the difference being significant for state 32223 for the 10-year duration (*p* = 0.04) and for all 4 states for the 20-year duration (*p* < 0.05).

### Regression results

The results of the mixed effects regressions are reported in Table 5. It shows that health state 32223 and 23232 receive lower utilities than state 22222, reflecting their higher impairments on several dimensions. Most demographic variables are not significant, except for one's own health rating, with healthier people trading off slightly more lifetime, and a marginal significance of income with larger incomes trading off less lifetime, but only in Model 1. Interestingly, the dummy for child perspective is positive and highly significant in Model 1, indicating uncorrected TTO utilities measured from the child perspective are on average 0.03 higher than TTO utilities measured from the own perspective. Model 2 illustrates that most results are similar for the corrected TTO utilities as for the uncorrected ones, with one notable exception. That is, the perspective dummy has become close to 0 and is no longer significant. This indicates that utilities are no longer valued higher from the child perspective than the adult perspective after correction for time preferences.

**Table 5:** Mixed effects regression on uncorrected and corrected TTO utilities

Variable	Model 1: Uncorrected TTO Coefficient (std. error)	Model 2: Corrected TTO Coefficient (std. error)
Constant	0.827 (0.293)***	0.836 (0.307)***
Age	− 0.0003 (0.002)	0.001 (0.002)
Male (reference: non-male)	0.050 (0.050)	0.058 (0.053)
EQVAS Own health today	− 0.004 (0.002)**	− 0.005 (0.002)**
Religious (reference: not religious)	− 0.026 (0.056)	− 0.009 (0.059)
Medium education (reference: low education)	− 0.058 (0.078)	− 0.061 (0.081)
High education (reference: low education)	− 0.029 (0.068)	− 0.026 (0.071)
Income (in categories)	0.030 (0.018)*	0.023 (0.018)
Has as at least one child (reference: no children)	− 0.000 (0.060)	0.006 (0.063)
Expected age of own death	− 0.000 (0.004)	− 0.000 (0.004)
Expected age of death 10y-old child	0.002 (0.003)	0.002 (0.003)
Dummy state 32111 (reference: 22222)	0.020 (0.014)	0.027 (0.016)
Dummy state 32223 (reference: 22222)	− 0.188 (0.014)***	− 0.181 (0.016) ***
Dummy state 23232 (reference: 22222)	− 0.231 (0.014)***	− 0.230 (0.016) ***
Dummy child perspective (reference: own perspective)	0.028 (0.010)***	− 0.001 (0.011)
Dummy 20y TTO (reference: 10y TTO)	0.010 (0.010)	0.005 (0.011)

Model 1: Log restricted likelihood: − 226.66. Wald Chi squared: 534.85, *p* < 0.001

Model 2: Log restricted likelihood: − 577.13. Wald Chi squared: 389.58, *p* < 0.001

***Significant at the 1%-level. **Significant at the 5%-level. *Significant at the 10%-level

## Discussion

This paper sought to investigate the effect of duration on the difference between TTO utilities measured from the adult’s own perspective and TTO utilities measured from a 10-year-old child’s perspective. In addition, we studied the effect of time preferences on both these TTO utilities and their difference. Although we found no significant differences between child and adult utilities in within-subjects tests, we did find significantly higher utilities for the child perspective than for the adult perspective after controlling for other variables in a mixed effects regression. Interestingly, correcting for time preferences removed this gap. Hence, the gap between child and adult TTO utilities may be partially driven by a difference in time preference between these perspectives. Extending the duration considered in TTO had no impact on utilities, neither from the adult perspective nor from the child perspective. The implication of these findings is that while a longer duration does not attribute to a smaller adult–child gap in TTO utilities (in line with the null-results reported in Lipman [21]), correcting for perspective-dependent time preferences does. Therefore, such a correction appears to be a worthwhile exercise.

The literature comparing adult and child TTO utilities shows mixed results, with some studies finding no systematic differences [14, 16, 17], but those studies that do, all report higher utilities for the child perspective than for the adult perspective [11, 13, 15]. The results of our study confirm the latter, although the gap is not substantial and only significant when controlling for other variables. To the best of our knowledge, there is only one previous study that compared child and adult perspective for time-preference-corrected TTO utilities, whose results are partly in line with our results [16]. Like us, they also found close to zero discounting and no significant differences between discounting from the two perspectives. However, in contrast to us, they reported no difference in health state utilities between the adult and child perspective. An explanation for this difference may be that their sample included Dutch respondents instead of UK respondents. Future work could directly test for country differences in perspective-specific time preferences by including both Dutch and UK respondents in their sample.

Our findings of a lack of discounting (on average) are worth discussing. Although these results confirm some previous studies [16, 43, 44, 54], other studies found higher discount rates[39, 48, 55–57].^2^ One explanation for the low amount of discounting in this and other recent studies is the use of the direct method. Because of its use of sequences, the sequence effect might induce respondents to prefer improving sequences over deteriorating sequences, which results in low, or even negative, discount rates [58, 59]. More specifically, we found 39.3% respondents were negative discounters for children and adults, while 25.3% discounted positively for both. However, similar findings of negative discounting have been present in other studies [16, 44, 54, 60, 61]. It is also worth noting that some popular methods ignore the possibility of negative discounting altogether [62], biasing estimates of discount rates upwards. Hence, it is advisable to replicate our study with an alternative time preference elicitation method that is less susceptible to a sequence effect (whilst still allowing negative time preference). It is unclear, however, if differences between child and adult perspectives would be similarly reduced with other methods.

Additionally, the method we used to elicit time preferences may also have captured other considerations. Respondents may think their ability to cope with a deteriorated health varies with their age [63]. For instance, they may reason that a poorer health state at the age of 80 is more acceptable than at the age of 50, being the result of the aging process. Alternatively, someone may argue that it is easier to cope with a health impairment at a young age than at an old age when they anticipate being more fragile. The answers given in the discounting task would then reflect a recognition of different life stage rather than discounting. We believe that exploring the influence of these alternative possibilities on time preference measurements is an interesting direction for future research.

Another limitation is that we did not perform validity checks for the time preference elicitation. Therefore, we cannot draw any conclusions about the robustness of our findings on this part. However, the time preference measurement was mainly included for supplementary analyses, while the main objective of this research was to test if the utility difference between child and adult perspective would be affected by incorporating a longer duration of 20 years instead of the standard 10 years. We encourage research in the future to further investigate the role of time preferences in cTTO valuation, including validity checks, such as test–retest reliability and an extensive test phase.

Compared to previous literature we observe a slightly higher percentage of weak and strict violations of dominance than in Lipman et al. [16], but comparable to Attema et al. [14]. This may have been caused by the relatively small differences between the health states in our study. In addition, we find more non-trading, more respondents without negative utilities, and less all-in trading than in Attema et al. [14], which can be attributed to the lack of very severe health states in our study. Our study is subject to a set of limitations. First, a limitation of our study is the use of video-interviews instead of physical interviews. This was unavoidable given the severe Covid-19 restrictions at the time of the data collection (autumn 2021). However, evidence suggests that video-interviews do not seriously decrease data quality [64, 65]. Second, the minimum admissible utility was higher for the 20-year duration (−0.5) than for the 10-year duration (−1). Here we had to make a trade-off between equal scales on the one hand and a realistic time horizon and identical lead-times on the other hand. For instance, if we wanted to maintain equal proportions of lead-time and time in impaired health, the maximum time horizon for states considered worse than dead would be 40 years. This would imply most years are spent in adulthood even in a child perspective, reducing differences with the adult perspective for WTD states. Moreover, the distortion caused by time preference would become even larger. Still, we do not think this has largely distorted our comparison, because not more than 2.6% of the responses was at the lower end of the scale for the 20-year duration, and this was only slightly higher than the maximum percentage of values equal to −1 (2.0%) for the 10-year duration. Furthermore, a robustness analysis where all utilities of the 10-year-task were censored at −0.5 generated similar results as the initial analysis. Still, we are unable to rule out the possibility that the difference in lead-time-to-disease-time ratio explains the similarity between the 10-year and 20-year TTO utilities.

A final limitation of this study is that it only provides a partial explanation of *why* utilities differ between adult and child perspectives. Earlier qualitative work has suggested a wide array of factors, not related to severity of health states, to influence valuation with child perspectives, e.g., Reckers-Droog et al. [66]. Besides our exclusively quantitative focus, the design used here allows concluding that time preferences differ between adult deciding for themselves or for 10-year-old children. As such, it is not clear if any effects are driven by differences between time preferences in deciding for self or other, or between deciding for adults or children. To identify such effects, a design like that of Lipman et al. [17] would be needed, who identified that the difference between adult and child perspectives appears mostly driven by the difference between deciding for other and deciding for self.

## Conclusion

We conclude that there is a small but significant discrepancy between uncorrected health state utilities elicited from the child and adult perspective in the EQ-VT protocol when controlling for other variables. In particular, respondents give up fewer life years in a TTO task when the child perspective is taken than when the adult (own) perspective is taken. This discrepancy is robust to the use of a longer gauge duration of 20 years, but it decreases after correcting for time preference. Therefore, similar health states do not seem to be valued systematically differently when they concern children than when they concern adults. Instead, individual, perspective-specific, time preferences may be partially driving the TTO responses.

## Data Availability

Data are available from the authors upon request.

## Appendix: Experimental instructions

### Introduction

Demographic questions are asked first, e.g., experience with serious illness, age, and gender. Interviewees then start this experiment by completing the regular EQ-5D-3L-Y assessment, which includes mobility, self-care, usual activities, pain/discomfort, and anxiety/depression. They will then be asked to overall evaluate how good or bad their health is today, from 0 to 100, where 0 represents the worst health, you can imagine and 100 means the best. A slider is used to indicate their choice. To be noted, the best or worst health states are not referred to being super rich or super poor, it is overall not related to material status. After that, they are assumed to be familiar with this system and move to the warmup section.

### Warmup section

#### Task 1

They are then asked to choose between two lives shown on the screen, A and B, which involves different health states and life duration. Respondents are informed that both lives would not change in any way as no medication or other possible treatment can extend or shorten the life duration (euthanasia). They can only think and choose from what they see on the screen, instead of thinking about how their choices could impact further tasks. After both lives, it is painless and immediate death. In the first example, life with a wheelchair, they start with choosing between 10 years in full health and 10 years in a wheelchair.

#### Task 2

A worse than death (WTD) or better than death (BTD) task will be invoked, based on respondents’ decision in task 1. That is, if the original answer indicates that the state is WTD, then a BTD choice is shown, otherwise, it is the WTD pathway after a BTD decision on the first task. The lead time trade-off method is expected to be explained in the WTD part for the respondents.

#### Task 3

Respondents are now asked to make a series of choices for themselves between Life A and B. They are reminded that at the end of the described period, it is immediate and painless death. The length of life (e.g., by changing your lifestyle or choosing euthanasia) cannot be changed. The quality of life (e.g., through pain relief or other medication) cannot be changed. Which life do you think is better? The first TTO task is then introduced and starts with letting the respondents choose between 10 years in full health and 10 years in a health state (21112). After that, they start with choosing between 10 years in full health and 10 years in another health state (32323).

### TTO part

#### Section 1

Respondents are now asked to make several choices for themselves between Life A and B. They are reminded that at the end of the described period, it is immediate and painless death. The length of life (e.g., by changing your lifestyle or choosing euthanasia) cannot be changed. The quality of life (e.g., through pain relief or other medication) cannot be changed. Which life do you think is better?

#### Section 2

Respondents are now asked to make several choices for a 10-year-old child between Life A and B. They are reminded that at the end of the described period, it is immediate and painless death. The length of life (e.g., by changing your lifestyle or choosing euthanasia) cannot be changed. The quality of life (e.g., through pain relief or other medication) cannot be changed. Which life do you think is better?

#### Section 3

Respondents are now asked to make several choices for themselves between Life A and B for 20 years. They are reminded that at the end of the described period, it is immediate and painless death. The length of life (e.g., by changing your lifestyle or choosing euthanasia) cannot be changed. The quality of life (e.g., through pain relief or other medication) cannot be changed. Which life do you think is better?

#### Section 4

Respondents are now asked to make several choices for a 10-year-old child between Life A and B for 20 years. They are reminded that at the end of the described period, it is immediate and painless death. The length of life (e.g., by changing your lifestyle or choosing euthanasia) cannot be changed. The quality of life (e.g., through pain relief or other medication) cannot be changed. Which life do you think is better?

### TTO feedback

Respondents are asked to answer whether they think the TTO tasks are easy to understand, whether is it easy to make the difference between those lives they are asked to think about, whether it is difficult to decide on the exact points where A and B are about the same. Their answers are indicated by five levels, strongly agree, agree, nether agree nor disagree, disagree, strongly disagree.

### Background questions

We then ask other demographic questions, including their religious belief, the highest level of education they have completed, total gross yearly income of their household, whether they have children and their life expectancy.

## Footnotes

1 The complete set of instructions is presented in Appendix A. The survey can be found here: https://referencepoints.shinyapps.io/GapDuration/.

2 However, other older studies also found little to no discounting on the aggregate level [54].
